# Rehabilitation of a Child with Neonatal Brachial Plexus Palsy: Case Report Described by Parents

**DOI:** 10.3390/children9091298

**Published:** 2022-08-26

**Authors:** Fátima Frade, Lurdes Neves, Fátima Florindo-Silva, Juan Gómez-Salgado, Lia Jacobsohn, João Frade

**Affiliations:** 1 Departamento de Enfermagem da Criança e do Jovem, Escola Superior de Enfermagem de Lisboa, Avenida Professor Egas Moniz, 1600-190 Lisboa, Portugal; 2 Centro de Administração e Políticas Públicas (CAPP), Instituto Superior de Ciências Sociais e Políticas da Universidade de Lisboa, Rua Almerindo Lessa, 1300-663 Lisbon, Portugal; 3 Escolher Brincar Terapia Ocupacional, Rua Professor Barbosa Soeiro 6, 4º Dto, 1600-598 Lisboa, Portugal; 4 Physiotherapy and Osteopathy Departments, Atlântica Health School, Universidade Atlantica, 2730-036 Barcarena, Portugal; 5 Serviço de Medicina Física e Reabilitação, Hospital Dona Estefânia-Centro Hospitalar Universitário Lisboa Central, 1169-045 Lisboa, Portugal; 6 Department of Sociology, Social Work and Public Health, Faculty of Labour Sciences, University of Huelva, 21007 Huelva, Spain; 7 Safety and Health Postgraduate Programme, Universidad Espíritu Santo, Guayaquil 092301, Ecuador; 8 Centro de Medicina de Reabilitação do Alcoitão, 2649-506 Alcabideche, Portugal; 9 Centre for Innovative Care and Health Technology (ciTechcare), Escola Superior de Saúde, Instituto Politécnico de Leiria, 2411-901 Leiria, Portugal; 10 Unit for Multidisciplinary Research in Biomedicine (UMIB), University of Porto, 4099-002 Porto, Portugal

**Keywords:** brachial plexus palsy, neonatal brachial plexus palsy, neonatal management, rehabilitation, physical therapy, occupational therapy, case report with parents

## Abstract

This paper presents a case report of a child with Neonatal Brachial Plexus Palsy on the right arm, with C5, C6, and C7 nerve injuries. The symptoms presented at birth and at the time of diagnosis were absence of movement in the right arm but with mobility of the fingers; internal rotation of the injured limb with elbow extension; active flexion of the wrist and fingers; and ulnar deviation of the hand. The rehabilitation plan followed the conservative approach and included different intervention strategies (passive and active mobilisation, kinesio tape, use of splints, bimanual stimulation, etc.) carried out by the occupational therapist and the physical therapist. The rehabilitation allowed the child to have a functional limb for daily activities, with bimanual motor integration and coordination; passive and active range of motion in the different joints except for pronation, sensibility, and maintained strength. In conclusion, it can be said that this case report describes a set of rehabilitation strategies that were used in the conservative treatment of a child with NBPP and the functional gains they allowed. Early intervention, parental involvement in the rehabilitation process, and continuous follow-up of the child favoured the prognosis and allowed the prevention of functional sequelae of the limb.

## 1. Introduction

Neonatal Brachial Plexus Palsy (NBPP) is a total or partial peripheral nerve injury which may affect C5–T1 cervical and thoracic roots, and which occurs during birth [1,2].

The injury occurs when there is stretching or rupture of myelin membranes or nerve trunk fibres or root avulsion. It can be classified according to the affected nerve complex, the degree of severity, and functionality. There is an upper trunk injury when the affected nerves are C5 and C6; a middle trunk injury when the affected nerve is C7; a lower trunk injury when the affected nerves are C8 and T1; and a complete injury when the affected nerves are C5–T1. The severity of the nerve injury is classified as avulsion injury when the nerve is torn; as neurotmesis injury when there is complete disruption of the axon and the connective tissue of the nerve; as axonotmesis injury when there is anatomical interruption of the axon, but no involvement of the connective tissue and nerve myelin; and finally, stretching of the nerve without interruption, which leads to a momentary blockage of the nerve–axon connection and recovers spontaneously. In the classification according to limb function, the NBPP lesion may be called Erb–Duchene syndrome or upper brachial plexus palsy (C5-C6), in which case shoulder abduction, shoulder external rotation, and elbow flexion are impaired, while hand function is maintained; Dejerine–Klumpke syndrome or lower brachial plexus palsy (C7-C8-T1), which affects hand and wrist function; and complete brachial plexus palsy (C5-C6-C7-T1), which compromises complete arm function. In the latter case, if associated with a sympathetic nerve injury, it is called Horner’s syndrome (C8-T1-T2), with the child presenting ptosis, miosis, and anhidrosis on the eye of the affected side [3,4,5,6].

NBPP often leads to dysfunction of the affected upper limb, which may be related to the motor and sensory functions of the child’s arm and may include loss of passive and active range of motion, sensory and muscle strength deficits, retractions, muscle contractures, and/or deformities and functional deficits [2].

Treatment options for NBPP are (1) conservative treatment, which includes intensive physical therapy and occupational therapy using techniques such as joint mobilisation, neurosensory motor stimulation, kinesio tape, electrostimulation, splint immobilisation, and constraint-induced movement therapy, among others, always including working in collaboration with families by teaching and training the family for continued intervention in a home context and medical intervention with botulinum toxin injection; and (2) non-conservative or surgical treatment, which may include primary and secondary surgeries. Primary surgeries are performed on children who have no spontaneous rehabilitation in the first three months of life, and secondary surgeries are performed on children who have significant functional limitations or functional deficits [5].

NBPP is complex and recovery patterns are not yet fully understood or predictable, so decisions about the best treatment remain a challenge [7].

Guidelines for the management of NBPP recommend (1) physical observation of the newborn with arm asymmetry or risk factors; (2) early referral to a multidisciplinary centre during the first month of age; (3) knowledge of pregnancy/delivery history and physical examination of the newborn at birth; (4) follow-up in multidisciplinary centres with therapists and neurosurgeons with expertise in NBPP; (5) physical therapy guided by a multidisciplinary team; (6) indication for nerve surgery if there is nerve avulsion or there are other surgical criteria resulting from the NBPP specialist team; and (7) data that should include Narakas classification, limb length, Active Movement Scale (AMS), and Brachial Plexus Outcome Measure (BPOM) 2 years after birth/surgery [6,8].

Studies have shown the effectiveness of using strategies such as Armeo robotic therapy, virtual reality, and plyometric training in the rehabilitation of children with Erb’s palsy, which can complement conventional therapy [9,10,11].

This case report describes a set of strategies that were used in the rehabilitation of a child with NBPP, the stages in the child’s development at which they were used, and the purpose of using these strategies.

The case of a child with the right arm affected by NBPP, with damage to the C5, C6 and C7 nerves, who presented in the first days of life with absence of movement in the right arm, internal rotation of the injured limb with elbow extension, active flexion of the wrist and fingers, and ulnar deviation of the hand is reported here. She underwent an intensive early rehabilitation process with conservative treatment, physical therapy, and occupational therapy.

In the rehabilitation of this girl, different intervention strategies were used (passive and active mobilisation, neurosensory motor stimulation, kinesio tape, use of splints, constraint-induced movement therapy of the healthy limb, bimanual stimulation, botulinum toxin, etc.).

This rehabilitation process allowed functional and sensory recovery, without resorting to the compensations typical of this type of condition (bugle sign and difficulty in elbow flexion), the integration of the upper limb in age-appropriate movements and activities, and the prevention of musculoskeletal deformities.

This work is relevant because it describes a set of strategies that were used in the conservative treatment for the rehabilitation of a child with NBPP. It highlights the adequacy of the strategies to the child’s development and the appearance of functions in the arm affected by the injury, as well as the functional gains that the child acquired in this rehabilitation process. It also highlights the importance of parental involvement in the rehabilitation process of the child with NBPP and the continued follow-up of the child.

This case study is organised according to the Case Reports Guidelines (CARE) following twelve steps: Title; Keywords; Abstract, Introduction, Patient Information; Clinical Finding; Chronology; Diagnostic Assessment; Therapeutic Intervention; Follow-Up and Outcomes; Discussion; and Patient Perspective [12]. The information contained in this work was reported by the parents and validated by the therapists accompanying the child.

## 2. Patient Information

This is the case of a Portuguese girl, Caucasian, currently 5 years old, with the right arm affected by NBPP. She was born on 10 March 2017 at 40 weeks + 3 days by forceps delivery, where there was shoulder dystocia. Good adaptation to extrauterine life, APGAR at birth 8 (1’), 9 (5’), and 10 (10’), weighing 4330 kg. After birth, she presented paralysis of the right upper limb with no movement in the arm and forearm, some mobility in the fingers, and no sensory response in the entire limb. There was no clavicle fracture. She developed neonatal jaundice on the second day of life and received phototherapy for 3 days, which was effective.

Pregnancy had been monitored, with some complications. The first trimester combined screening result showed a risk for trisomy 21. The amniocentesis was negative. In the first trimester, the pregnant woman had a slight placental abruption that reverted with rest, and at the end of pregnancy, she developed hepatic cholestasis. The mother had no history of diabetes.

## 3. Clinical Findings

Upon physical examination after birth, the child had the right upper limb along the body in internal rotation and with the elbow extended, the hand with active flexion of the wrist and in ulnar deviation, and the fingers flexed. In the evaluation of primitive reflexes, the asymmetric moro reflex and the asymmetric cervical tonic reflex were absent, and there was no response to tactile information. She was diagnosed in the maternity ward with Erb–Duchene Palsy or Right Upper Brachial Plexus Palsy (C5-C6) and was then referred for physical medicine and rehabilitation.

Recovery of function of the affected limb was monitored over time, essentially through physical observation (physical examination), at key moments, which predicted the evolution and prognosis of NBPP recovery.

Physical assessment, which included observation of joint amplitude, assessment of muscle strength, posture and movement patterns, symmetry and alignment, muscle com-pressions, contractures and deformities, shoulder girdle stability and background movements of posture and global motor coordination (rotation, dissociation of movements, etc.) was performed.

Sensory assessment was carried out by observing tactile, proprioceptive, vestibular and visual sensations, bilateral motor integration, and bilateral motor coordination.

## 4. Timeline

Follow-up of the child presented chronologically:

*10 March 2017 (aged 1 day)*. Diagnosis of NBPP (Erb–Duchene Palsy or Right Upper Brachial Plexus Palsy (C5-C6)) was made by the paediatrician from the maternity area.

*20 March 2017 (aged 10 days)*. Physiatry consultation in the physical medicine and rehabilitation service, which prescribed intensive physical therapy and occupational therapy. The assessment by the physiatrist was carried out very closely during the physical therapy or occupational therapy session; whenever there was any significant recovery, the therapists asked the physiatrist to observe the child during the session. Consultations were formally held every 3 months (at 3, 6, 9, 12, 15, 18 months), from 18 months onwards, which was when intensive care ended, and every 6 months, which are still maintained.

*From March 2017 to September 2018 (0 to 18 months)*. Start of physical therapy three times a week and occupational therapy twice a week.

*End of March 2017 (First month of life)*. Plastic and reconstructive surgery consultation, the aim of which was to assess the need for surgical intervention for reconstruction of the affected nerves. Surgical evaluation was performed in the first month of life (26 March 2017), and then every 3 months until 12 months of age. After this age, she was evaluated every 6 months and after two years, annually.

*By June 2017 (3 months)*. The child was examined by the physiatrist and surgeon to check whether she had elbow flexion against gravity. At three months, the child did not have elbow flexion against gravity, but there was recovery in relation to shoulder extension and elbow flexion in favour of gravity, so it was decided to wait until 6 months to decide on the need for surgical intervention; electro-stimulation (16 Hz, 200 Us, 20 min a day on the biceps muscle) was prescribed by the physiatrist. Electromyography was performed to study the motor potential of the right ulnar nerve for a possible transfer of the ulnar nerve to the biceps brachii muscle.

*By July 2017 (4.5 months)*. Elbow flexion against gravity appeared.

*By September 2017 (6 months)*. Follow-up of plastic and reconstructive surgery deemed that surgical intervention was not considered necessary. Occupational therapy and physical therapy were maintained, and one-to-one hydrotherapy commenced with a specialist therapist once a week. Magnetic Resonance Imaging (MRI) of the right upper limb was performed for staging and exploration of shoulder function.

*By December 2017 (9 months)*. At the plastic and reconstructive surgery consultation, it was noted that the child was progressing well, with normal biceps function, but with a slight external rotation deficit, so botulinum toxin in the subscapularis muscle was proposed to the physiatrist, who decided to wait until 12 months.

*By April 2018 (13 months)*. With deficit in external rotation and supination, the patient underwent botulinum toxin injection in the subscapularis and teres major muscles and the pronator teres, and weekly intensive therapies were maintained (3 days of physical therapy and 2 days of occupational therapy), also adding electrostimulation 16 Hz, 200 Us, 20 min a day in the supinator and extensor muscles.

*By June 2018 (15 months)*. External rotation and supination appeared.

*By September 2018 (18 months)*. Plastic and Reconstructive Surgery consultation: surgery did not actively assess the limits of external rotation and supination, but passively, there did not seem to be a deficit that justified subscapularis release by surgery. Intensive therapy was discontinued, and physical activities (swimming and ballet) were initiated to allow maximum development of the motor skills.

*By March 2019 (2 years)*. Physical medicine and rehabilitation consultation: physical activity was recommended, and no indication for surgery was made in the plastic and reconstructive surgery consultation.

*By March 2020 (3 years).* Due to the COVID-19 pandemic, some activities were suspended, such as swimming and ballet (as the institution did not guarantee safety conditions).

The lack of physical activity and the lack of parental time to accompany her in activities at home (due to the birth of her brother) led to the appearance of patterns/compensations and difficulty in performing full pronation, and when not in use, the arm remained in the supine position.

To promote pronation, kinesio tape was used, as well as activities such as wheelbarrow, play dough, puzzles, etc. At the plastic and reconstructive surgery consultation, the accompanying physician considered that, given the child’s favourable evolution, the criteria for nerve surgery were not met.

*By September 2021 (4 years).* MRI of the right shoulder joint was performed to assess the need for referral to paediatric orthopaedics and the eventual need for orthopaedic surgery.

*By October 2021 (4 years).* In the physical medicine and rehabilitation consultation, the physiatrist discarded the need for referral to orthopaedics at this stage. At the plastic and reconstructive surgery consultation, nerve surgery was not considered necessary. The MRI of the right shoulder did not reveal significant changes, so annual monitoring by the plastic and reconstructive surgery consultation was maintained. As for the child’s growth and development, she has evolved in a healthy manner.

## 5. Diagnostic Assessment

The diagnosis and staging of NBPP was essentially made through physical observation, considering the evolution and/or recovery of motor and sensory function. However, at key moments (3 months and 6 months), it was necessary to resort to diagnostic tests to decide on the most appropriate therapeutic option.

Thus, at 3 months, an electromyogram (EMG) was performed, where the nerve conduction velocity of the right ulnar nerve was studied, showing a good potential amplitude. The EMG report revealed a moderate neurogenic tracing of the deltoids and biceps brachii, more impoverished in the arm and axonal lesion of the C5-C6 roots (upper primary trunk) with reinnervation, but with evident loss of motor units in the cutaneous muscle territory. Motor potential of the ulnar nerve with acceptable amplitude was observed, with low sensory potential. The electromyogram was performed to assess the ulnar nerve potential, since if there was no elbow flexion, one of the surgeries that could be performed was the transfer of the ulnar nerve to the nerve of the biceps muscle.

At 6 months, an MRI of the right upper limb was performed, which revealed, at the level of the brachial plexus, traumatic thickening/neuroma/meningocele of the C6-C7-C8-T1 roots. Signal alterations persisted until the formation of the distal trunks, dividing the nerve endings differently. As for the muscular apparatus, the right shoulder girdle, in relation to the left, did not show atrophy or significant asymmetry of the muscular planes. Regarding the osteoarticular apparatus, humeral glenoid showed retroversion with respect to the right glenoid (right glenoid—20 degrees; left glenoid—11 degrees). The hyposignal alteration of the posterior margin of the cartilaginous glenoid reflected incipient dysplasia. The purpose of the MRI performed at 6 months was to check for alterations in the shoulder girdle joint (e.g., glenohumeral subluxation), as this sequela can compromise the entire function of the arm and early identification can prevent its recurrence.

At 4 years of age, an MRI of the right shoulder was performed in which there was a slight superior subluxation and a right posterior glenohumeral subluxation; morphology of the glenoid cavity relatively was maintained, with only a discrete flattening, but without signs of manifest dysplasia; right humeral head had preserved morphology; and the muscles of the shoulder girdle had reasonable volume, with the signal maintained except for the impossibility of comparison with the contralateral arm. No joint effusion was observed. This MRI was performed in order to assess the function of the shoulder girdle and prevent the sequelae.

Imaging tests (MRI) were performed under sedation. The electromyogram was performed without sedation, with a needle electrode, as the child had decreased sensitivity in the injured arm, experienced no discomfort, and remained calm throughout the procedure. The parents were a little anxious about the performance of these invasive diagnostic tests for the child.

## 6. Therapeutic Interventions

Interventions for recovery from NBPP were incorporated into physical therapy sessions held 3 days a week and occupational therapy sessions held 2 days a week, continuing in this intensive manner until 18 months. Sessions lasted 45’ to 60’, but the child’s tolerance was taken into account. It is important to note that many of the intervention strategies carried out in the sessions were taught to the parents in a collaborative way, so that they could reproduce these exercises at home at different times of the day, thus increasing the rehabilitation potential of the child with NBPP.

This child underwent an intensive early rehabilitation process based on neurodevelopmental techniques (neurological, sensory, and motor development), musculoskeletal techniques, and individualised muscle-strengthening strategies. Different intervention strategies were incorporated considering the child’s development and the emergence of more functions in the arm affected by the injury.

**Figure 1:**
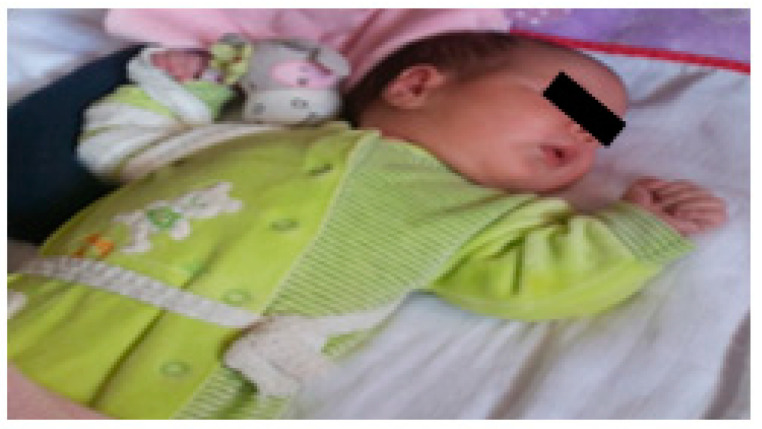
Twist strap.

**Figure 2:**
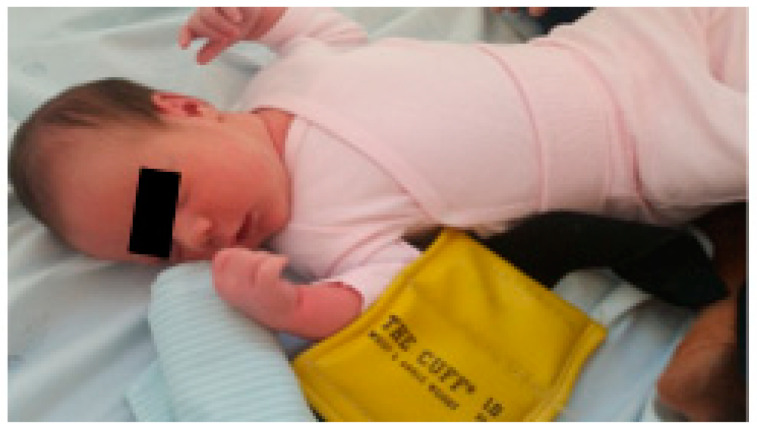
Weight to maintain posture.

Physical therapy and occupational therapy started at 11 days of age. In a first phase, parents were taught the importance of maintaining proper posture of the limb throughout the day to keep joint range of motion and prevent postural patterns, anteroposterior capsule retraction, contractures, and deformities; efforts were made to keep the affected arm in external rotation with the help of a torsion bandage or a weight (Figure 1 and Figure 2). These strategies were used when the child was at rest, with the aim of integrating the limb into a symmetrical posture and pattern appropriate to the stage of the child’s development by physiologically aligning the shoulder, arm, forearm, and hand joints, preventing expected retractions/contractures and deformities (e.g., shoulder dysplasia) and promoting/facilitating the most normal developmental sequence possible.

**Figure 3:**
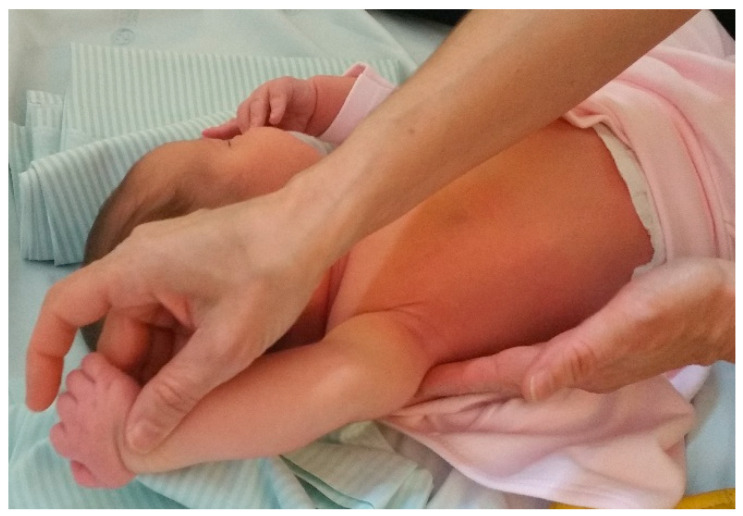
Passive mobilisation of shoulder flexion, with scapula support.

**Figure 4:**
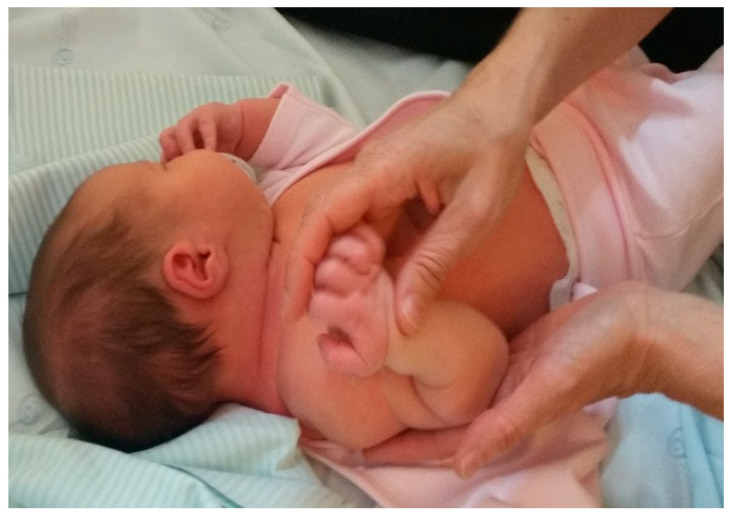
Passive mobilisation, elbow flexion.

Passive mobilisations of the affected limb were taught to the parents, who reproduced them several times throughout the day. These were essential to avoid joint stiffness and maintain joint range; they had to be performed gently and with each compromised joint. These passive mobilisations included shoulder external rotation, shoulder flexion, elbow flexion and extension, forearm supination, and wrist and finger extension. These mobilisations were performed on this child in sets of 10 repetitions of the movement, 4 to 5 times a day. Passive mobilisations (Figure 3 and Figure 4), in addition to preserving the functional capacity of the joints, are an important source of proprioceptive stimulation for the integration and recovery of the affected nerves.

In passive mobilisations, one of the most important movements is the external rotation of the shoulder with the scapula stabilised to prevent contracture in the internal rotation of the shoulder and shoulder deformity (glenohumeral dysplasia, as this is the problem that generates most disability in NBPP). Performing this movement several times a day increased shoulder flexibility and prevented shoulder deformity and shortening and atrophy of the subscapularis muscle, thus delaying glenohumeral deformity. Passive mobilisations were very important in the first three months of life, as they took into account the child’s development and the inability to stimulate with intentional activities (as its development did not yet allow it), but were maintained throughout the first and second years of life.

**Figure 5:**
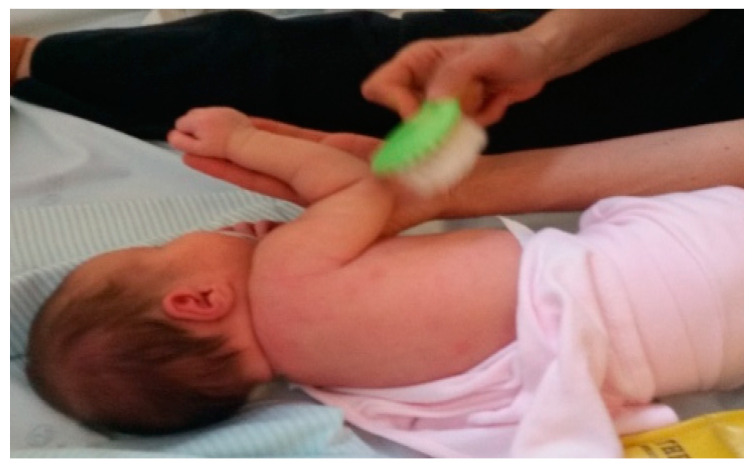
Sensory stimulation with a soft brush.

In addition to passive mobilisations, tactile, and proprioceptive sensitivity was also stimulated (Figure 5) in the affected arm through tactile stimulation with materials of different textures, sizes, shapes, temperatures, vibrations (soft brush, electric toothbrush, feathers, bath bags, ice, etc.), and also by sucking on the thumb of the hand of the injured limb. This strategy provides sensorimotor input and promotes oculomotor coordination and central integration of the affected limb, increasing sensory perception of the injured arm as an integral part of the body.

Following the child’s neurosensory motor development, from the age of 3 months onwards, active mobilisation stimulation of the midline of the body began to be incorporated as a facilitator of the integration and incorporation of the affected limb. Thus, loading/proprioception activities were stimulated in the prone position, and the midline in the supine and lateral positions.

**Figure 6:**
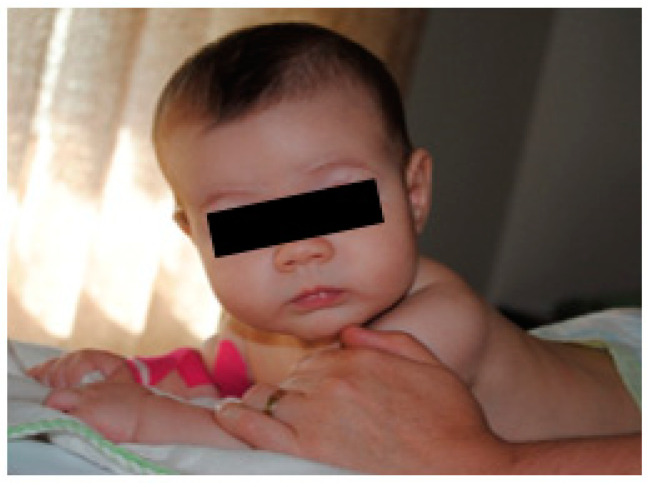
Prone position.

**Figure 7:**
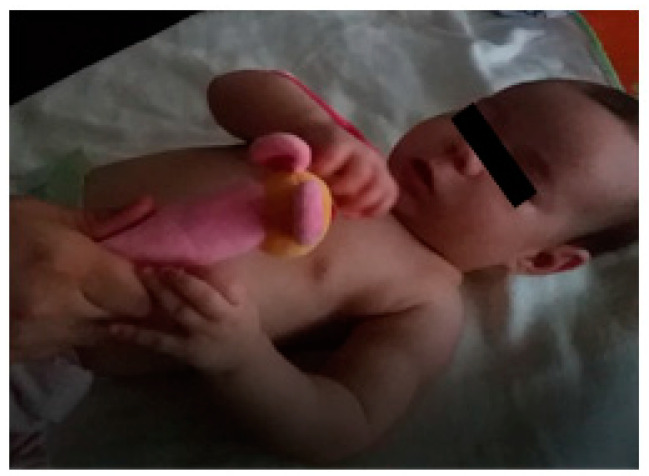
Supine position.

The prone position, with the arms supported, allowed the loading of the whole limb and the activation of the scapula stabilisers to be strengthened. Hand, forearm, and shoulder in neutral and prone positions are visible in Figure 6; in the supine position, the midline allowed the simultaneous use of both arms and, therefore, bilateral motor integration (bimanual) (Figure 7); lateral decubitus allowed the work on the midline in favour of or against gravity, uncovering, and exploring the hands.

From the age of 3 months onwards, manipulative function was greatly stimulated to accompany the child’s own development, as at this stage it is very important for the child to use vision as a way of reinforcing somatosensory information (tactile and proprioceptive) and to integrate the limb in bimanual activity (see both ends) to develop the function.

These activities provide tactile and proprioceptive sensory information that serves as a basis for voluntary use, preceded by motor activity of the injured limb. It is very important to take advantage of neurosensory motor development, postural control, loading, weight transfers, rotations, movements, and especially manual activity to integrate the injured limb into all activities.

**Figure 8:**
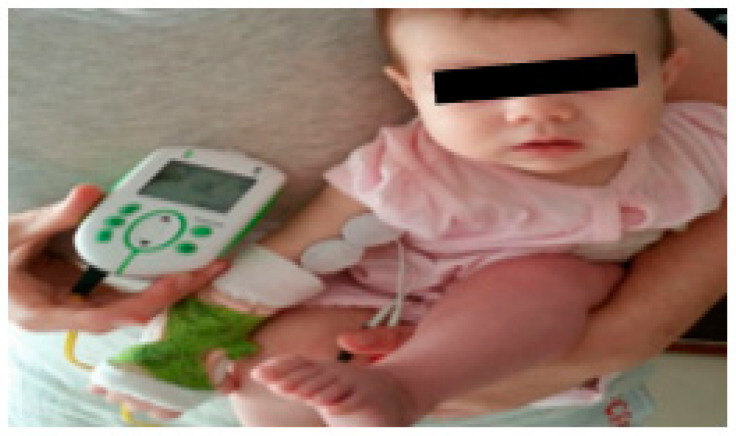
Electrical stimulation of the biceps and posterior splinting of the hand and wrist.

At three months of age, as this child still did not have elbow flexion against gravity, another strategy was added to improve rehabilitation, which complemented the intentional active mobilisations, this being biceps electrostimulation (Figure 8), with the intensity of 16 Hz, 200 Us, 20 min a day. At a more advanced phase (15 to 18 months), 16 Hz, 200 Us, 20 min a day electrostimulation was also applied to the supinator and extensor muscles. Electrostimulation prevents muscle atrophy and increases muscle strength.

Between 4 and 5 months of age, active loading was stimulated, in the prone position, with greater extension of the upper trunk and functional use of elbow extension/flexion and hand support.

This child initially had wrist flexion, so a palmar splint for the wrist and hand was applied for short periods throughout the day and night. The purpose of using this splint was to maintain correct wrist alignment, promote wrist extension, and prevent/reduce flexor shortening/contractures. Its use was closely monitored, as when the child becomes more active, its use may be inappropriate because it impedes movement.

**Figure 9:**
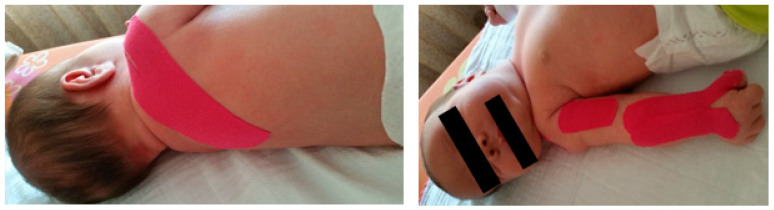
Kinesio Tape applied to different muscle groups.

Another strategy used throughout the rehabilitation process and still used today to promote specific muscle work or facilitate a postural pattern was the application of kinesio tape on different muscle groups of the affected parts for 3–4 days (Figure 9). It consists of the application of elastic bands that help to activate the affected muscle groups.

From 8 months onwards, work was carried out on upper limb weight loading, transition from sitting to the four-legged position and vice versa, and finally, crawling.

**Figure 10:**
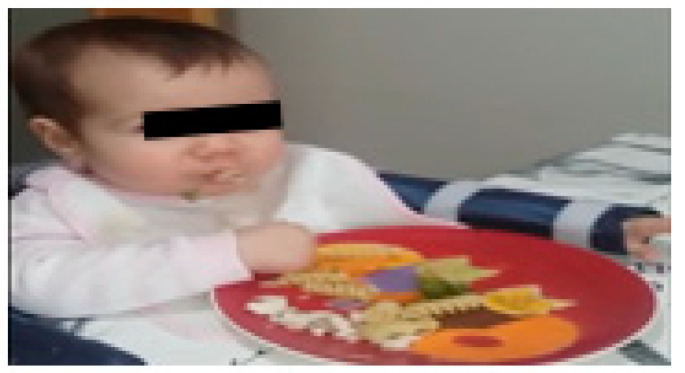
Constraint-induced movement therapy.

Between 9 and 12 months, as the child showed increasing integration of the limb, demonstrated by the acquisition of manual motor skills and a certain autonomy in activities such as feeding using the hands, the parents incorporated complementary feeding through Baby-Led Weaning (BLW) to promote the intensity of sensorimotor experiences and thus facilitate the development of a flexor pattern, manipulative skills, and interaction through functional use (Figure 10). This intervention, with a maximum duration of 2 h a day, allowed, stimulated, and promoted more frequent use of the injured limb, enabling an increase in the frequency and intensity of sensorimotor experiences and thus facilitating the development of a flexor pattern, manipulative skills, and interaction through functional use.

At 11–12 months, standing and gait position were not encouraged, in order to continue working on upper limb activities under load.

At 13 months, the child received botulinum toxin in the subscapularis, teres major, and pronator teres because she presented deficits in external rotation and supination. The aim of this strategy was to temporarily inhibit the function of these muscles, so that the weaker muscles could be activated and strengthened. Botulinum toxin is a toxin that blocks nerve conduction in the muscle for a variable period of 2 to 6 months, so after administration there was a window of opportunity to work on the deficient movements both passively and actively, in this case with external rotation and supination.

As the child developed, skills such as rolling, crawling, sitting, and hand manipulative skills were encouraged to achieve maximum mobility of the injured limb and integrate it into functional daily life activities.

**Figure 11:**
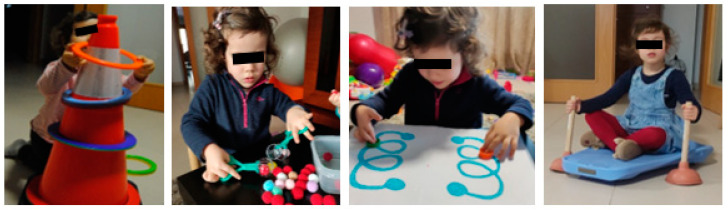
Bilateral integration of the injured limb.

Most daily life activities are bimanual, so one of the rehabilitation strategies used in the rehabilitation of the child with NBPP was bilateral integration of the injured limb, for which the child performed activities that required the use of both hands. She performed bimanual activities (Figure 11) with the aim of gaining dexterity and skills in the most weakened muscle groups through use and repetition.

**Figure 12:**
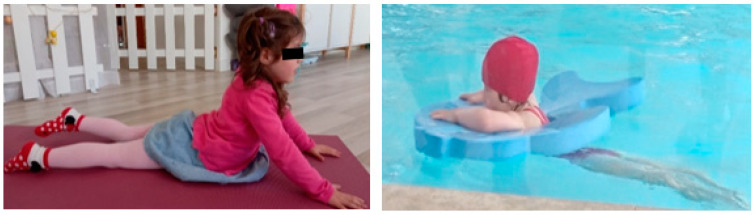
Leisure and sports activities (yoga and swimming).

Leisure and sports activities (Figure 12) are activities that the child with NBPP performed frequently, namely, swimming twice a week, yoga once a week, and recreational activities that could be performed at home or in playgrounds, such as climbing, cycling, swimming, etc.

Swimming is an activity that favours the use of the injured limb and avoids excessive effort, so while the child moved in the water to control her body, she learned to use muscle groups and perform joint movements that would be more difficult out of the water. The positions used in yoga stretch the muscles and increase flexibility.

**Figure 13:**
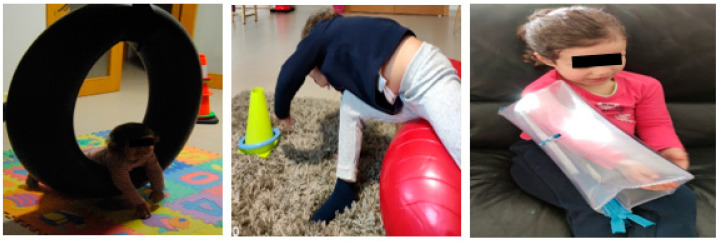
Stretching and use of Urias^®^ splint.

In addition to the strategies mentioned above, massage of the affected limb, stretching, and Urias^®^ splinting were also used (Figure 13). Massage prevents or reduces contractures that develop in the most active muscles and can cause restriction of arm movement. Urias^®^ splinting and stretching promote muscle relaxation and deep tactile information/proprioception that prevent contractures and facilitate harmonious working of the muscle groups.

**Figure 14:**
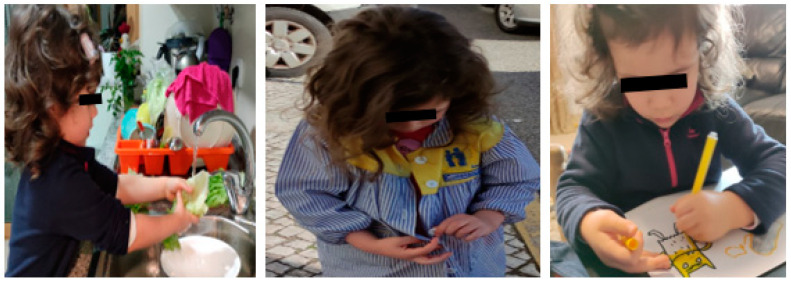
Personal autonomy.

Finally, the promotion of personal autonomy was encouraged from a very early age, as most daily activities involve the acquisition of manual skills in both upper limbs, i.e., dressing oneself, eating, bathing, brushing teeth, combing hair, cutting, drawing, writing, doing puzzles, etc. (Figure 14).

## 7. Follow-Up and Outcomes

The child with NBPP presented in this case report underwent an early-onset conservative rehabilitation process, without the need for reconstructive surgery of the injured nerves, where different intervention strategies listed above were implemented.

As a result of these interventions, a currently functional limb was achieved for a child with the right upper limb affected by NBPP. At present, the child has a functional limb, a protruding shoulder, full passive range of motion in the different joints, except for pronation (15°), and is active with the exception of forearm pronation, in which she assumes the neutral position, compensating the lack of active range with internal rotation of the shoulder. She has sensibility and strength, as well as adequate bimanual motor coordination. As expected, the limb is not dominant, and as such, a secondary support role is assumed in many unilateral activities.

**Figure 15:**
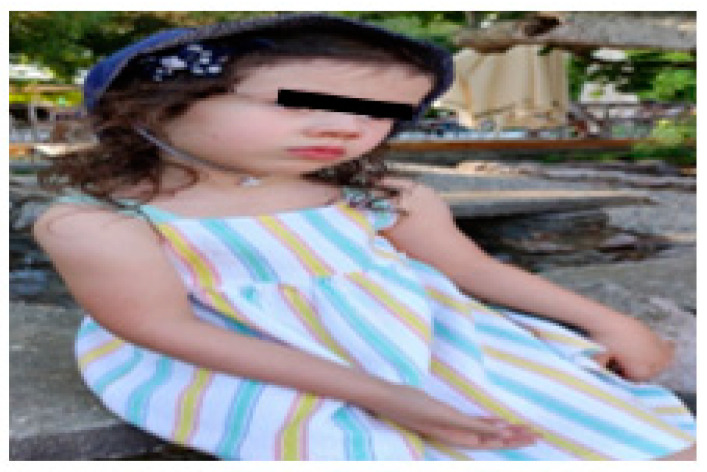
Injured limb at rest.

The shoulder is slightly protruded, with the scapula slightly abducted and in external rotation. When in motion, especially while running, the injured limb has the elbow in extension, but with dissociation of movements, which favours balance during running. In the sitting position, when performing unilateral activities, she is sometimes still necessary to remind her of the need to place the arm on the table, and the forearm tends to be in slight supination, with the hand in slight ulnar deviation (Figure 15).

**Figure 16:**
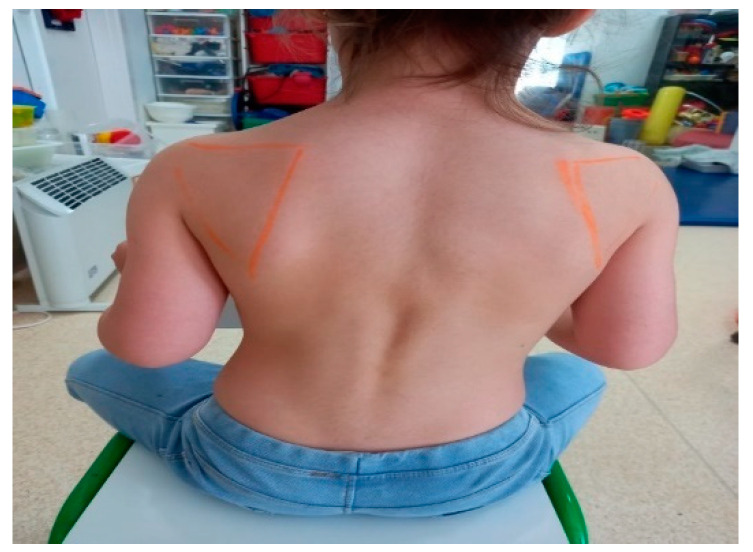
Protrusion of the scapula.

The limb is functional and integrated. It participates in bilateral activities spontaneously, substituting the limitation in pronation for internal rotation of the shoulder. In unilateral activities, it is used as a supporting limb, as is the non-dominant limb at this age. In terms of symmetry and alignment, the scapula protrudes slightly (Figure 16).

The photographs presented here were taken by the parents at home while working on their daughter’s rehabilitation.

## 8. Discussion

The present study represents the rehabilitation of a child with the right arm affected by NBPP, whose rehabilitation included conservative treatment, with follow-up by physical therapy, occupational therapy, physiatrist, and neurosurgery.

This child did not require surgical intervention. In the conservative rehabilitation process, physical therapy or occupational therapy strategies were used, which considered the child’s neurosensory motor development using passive mobilisations, active mobilisations, electrostimulation, botulinum toxin, kinesio tape, constraint-induced movement therapy, use of splints and torsion bandages, bilateral limb integration, recreational and sport activities, massage, stretching, and relaxation splints (Urias^®^ splint). Personal autonomy in daily life activities was promoted, and the development of a collaborative work with parents, where, through their empowerment, they became active agents in the child’s rehabilitation.

The early intervention of physical therapy and occupational therapy was relevant in the rehabilitation process, as it allowed the good restoration of function, the development of the injured limb, and the healthy development of the child. Other studies corroborate the importance of starting the rehabilitation process as early as possible, as it prevents the onset of neuromuscular skeletal and sensorimotor alterations, adhesions, retractions, contractures, and deformities and, consequently, sensory and motor changes, such as muscle shortening, decreased range of motion, and appearance of sequelae [13,14].

The development of a model of care in collaboration with parents, in which they were taught some rehabilitation strategies that could be reproduced at home several times a day such as passive mobilisations and active mobilisations using games, had a great impact on the rehabilitation of this child. Other authors demonstrate that movement training programmes can be performed at home by parents as a way to complement the rehabilitation of children with NBPP, as these programmes aim at range of motion and increased muscle strength [14,15,16].

Passive mobilisations, postural correction, and sensory stimulation were the interventions used from the beginning of the rehabilitation process because in the first months of life, given the child’s development, they were the most appropriate. This facilitated the preservation of the functional capacity of the joints and provided an important proprioceptive stimulus for integration and recovery from the injury, thus preventing sequelae. Several authors state that the pillars of the treatment of NBPP include postural correction of the affected limb, passive mobilisations, muscle percussion and massage, and active mobilisations, which should take into account the neurodevelopment of the child [4,5,14].

The use of thermoplastic splints for controlled periods of time allowed for control of wrist flexion in this child, promoting proper joint alignment, wrist extension, and prevention of contractures that could limit wrist and hand function. The use of splints for controlled periods of time, and especially at night, allowed the child to have the limb free during the day for active movement. A study evaluating the results of the use of orthoses in children with NBPP states that their use for recommended periods of time may have a positive effect on balanced growth of the affected limb, muscle function, and long-term outcome prognosis in these children [14,17].

In this case, electrostimulation provided significant improvements in activation, strengthening the muscles of the affected limb, specifically elbow flexion and wrist extension. Several studies have shown that electrostimulation in NBPP rehabilitation should be used as a complement to training in the use of the upper limb affected by the injury, as it accelerates nerve regeneration, improves muscle strength, and prevents muscle shortening, flaccidity, joint deformities, and muscle contractures [18,19,20].

The use of kinesio tape in this child allowed a better alignment of bone structures and favoured the activation of muscle function. Walsh, in a study conducted with children with NBPP who used kinesio tape in their rehabilitation process, concluded that the use of this ally of active exercises facilitated functional, muscle, and bone changes [21].

Constraint-induced movement therapy was also used, which aimed to enhance use and develop skills, dexterity, and strength of the affected limb. The use of constraint-induced movement therapy, such as splints, was limited to controlled periods of time. Different studies report that the use of constraint-induced movement therapy in the performance of daily functions improves the motor function of the affected limb through repetition training [4,22].

To enable the acquisition of external rotation and supination movement, botulinum toxin was administered to this child. The botulinum toxin temporarily paralysed the function of the subscapularis, teres major, and pronator teres muscles, so that the muscles affected by the injury could be worked on to gain strength and functionality. García Ron et al. report in their article that botulinum toxin can be used as a complementary treatment to physical therapies and surgical treatment of NBPP, proving to be safe and effective [23].

Bilateral integration of the injured limb, recreational and sport activities, and promotion of personal autonomy were some of the activities carried out with the child after the end of intensive therapies (after 18 months), and these were aimed at maintaining the motor and sensory function of the affected limb, promoting the gain of awareness of the arm integrated into the body. Yanes Sierra et al. refer that in older children with NBPPP, it is important to promote physical activity, such as hydrotherapy, swimming, and others, as well as the use of games that increase body awareness of the arm and occupational therapy that follows the motor development of the child [14].

In conclusion, it can be said that this case report describes a set of rehabilitation strategies that were used in the conservative treatment of a child with NBPP and the functional gains they allowed. The rehabilitation strategies used were implemented taking into account the developmental stage of the child and the functional gains that the child was acquiring in the injured limb.

In this child, the criterion for not requiring nerve surgery was the fact that she presented recovery with conservative treatment, i.e., the acquisition of elbow flexion against gravity (between 3 and 6 months) and external rotation (between 12 and 15 months).

Early intervention, which, in the case of this child, started at 11 days of life, was decisive in increasing the rehabilitation potential of the injured limb, preventing musculoskeletal sequelae, thus stimulating the integration of the injured limb into sensorineural motor development and daily life activities.

Continuous monitoring of the child was very important, as it allowed us to avoid sequelae, which can lead to changes in the symmetry and functionality of the injured limb.

The involvement of the family in the rehabilitation process was fundamental; the parents reproduced at home the exercises taught in the therapeutic sessions, and the repetition of the same exercise throughout the day increased the rehabilitation potential and the functionality of the injured limb.

As limitations of this study, it is worth mentioning the fact that it is a case study, so the results obtained cannot be extended to the population of children with NBPP; furthermore, assessment scales, such as the Active Movement Scale (AMS) and the Modified Mallet Scale [24], were not used to monitor the evolution of the affected limb of this child, as these instruments are not translated and adapted to the Portuguese culture and population.

Thus, the need for research on rehabilitation strategies used in children with NBPP is identified, as well as the validation of scales to assess the evolution of children with NBPP for the Portuguese population.

## 9. Patient Perspective

Our case report concerns a 5-year-old girl. Currently, her perspective on the injury is related to the way she perceives the injured arm: she refers to it as “the arm weaker than the other one”, and that is why she has to perform exercises to “make it stronger”. Given the age of this child, we consider relevant the perspective of the parents, who were actively involved in their daughter’s rehabilitation.

From the parents’ point of view, the child has this perspective because she has to perform daily activities/exercises that allow that arm to be as agile as the other. To date, they do not feel that the child feels in any way limited by the presence of the NBPP; the use of the left arm as the dominant one is considered by the child as natural, as it has always been this way.

Regarding the rehabilitation of their daughter, the parents describe the process as intense and the impact of the diagnosis on their family as enormous. They were first-time parents of a child who acquired an injury during childbirth. They comment on the unpredictability of the rehabilitation process and on being involved in it. These factors generated feelings of fear, insecurity, and helplessness, as they feared that they would not be able to reproduce what they had been taught by health professionals, or whether they would do it effectively.

The parents took the child to the therapy sessions and attended the sessions, where they were taught the different rehabilitation strategies to be reproduced at home (all the used strategies have been included in this work). In the intensive rehabilitation period, these strategies were reproduced six to eight times a day, depending on the child’s tolerance, five days a week. The weekend was assumed to be a rest period for the parents and the child. After the intensive rehabilitation period and up to now, every moment is used to promote the activity of the injured limb, while playing, bathing, feeding, etc.

They consider that the training of parents is fundamental in the rehabilitation process of children with BPPN, but it is also necessary to take care of these parents with psychological support, as they are in a situation of great emotional vulnerability.

These parents did not receive psychological support. Instead, they shared their fears and how they felt during the whole process with the therapists and the physiatrist, but psychological support was never provided. They ended up receiving this support through the health professionals who accompanied their daughter and from family and friends. Concern for their daughter persists, as they fear that there may be sequelae that could affect her quality of life.

## Data Availability

All data are available within this article.
